# In Silico Design of a Peptidomimetic Carrier for Levodopa

**Published:** 2011-03

**Authors:** A. Banerjee, A. Yadav

**Affiliations:** *Department of Chemistry, University Institute of Engineering and Technology, CSJM University, Kanpur, India*

**Keywords:** levodopa, brain bioavailability, carrier, peptidomimetic, lipophilic

## Abstract

Ab initio molecular orbital calculations at the Hartree Fock level utilizing 6-31G basis set have been performed on small cyclic peptides and peptidomimetic compounds to explore their utility as carriers of levodopa (L-3,4-dihydroxyphenylalanine) to enhance its brain bioavailability. A cyclic peptidomimetic compound with hydrophobic CH_2_NH backbone is suggested as possible carrier. This carrier is predicted to efficiently carry Levodopa held by non covalent interactions encompassed in its cyclic backbone without chances of expulsion before delivery inside brain. Carrier is expected to undergo passive diffusion alongwith the drug held inside. Once inside the brain, drug may be delivered enzymatically or non enzymatically.

## INTRODUCTION

Levodopa (L-Dopa) is currently main therapeutic drug available for symptomatic treatment of Parkinson’s disease (1). However, there are several adverse side effects associated with long-term levodopa therapy (2). Levodopa is generally administered in combination with a peripheral decarboxylase inhibitor like carbidopa to maximize the amount of levodopa available to enter brain (3). It not only increases the bioavailability of levodopa but at the same time reduces adverse effects due to peripheral decarboxylation like nausea, vomiting and hypotension. Long term usage of levodopa leads to motor fluctuations, dyskinesias and neuropsychiatric effects (4). Levodopa can be administered transdermally in the form of Levodopa-ethyl-ester (5). The aim of this work is to recommend an appropriate carrier in the form of prodrug for levodopa to enhance its bioavailability. The main reason why levodopa came into existence was the ability of this compound to cross blood-brain-barrier (BBB) using same carrier as that of phenylalanine (6). Inside brain it is decarboxylated and available as dopamine (as needed!). Dopamine then alleviates symptoms of Parkinson’s disease.

Although levodopa is clinically used; its brain exposure has always been a point of concern and discussion for over a decade (7). Levodopa has been presumed to be transported across BBB using large neutral amino acids (LNAA’s) transporter system L. It has to compete with other AA’s to be transported (8). In addition Levodopa also undergoes passive diffusion across BBB (9). It has been observed that protein rich meal lowers Levodopa concentration in brain as the competition for transporter increases and saturation levels are observed (10). Recent research work by Hawkins and co-workers has shown the existence of a Na^+^ dependent transport system on abluminal side of BBB that transports AA’s in opposite direction that is, from brain to blood against concentration gradient (11). This phenomenon has been cited as one of the causes of decreased brain exposure of Levodopa. To avoid a saturable mechanism for Levodopa, metabolically active glutathione analogs have been used as carrier for Levodopa brain delivery (12). A lipophilic carrier needs to be used for passive diffusion. Such examples include polymer nanoparticles loaded with Curcumin to enhance brain exposure of Curcumin for Alzheimer’s disease patients (13). Peptides can cross BBB in both ways that is, through transmembrane diffusion, a non saturable mechanism depending on lipophilicity of peptide or they may be transported by saturable systems across BBB. For example, halogenated enkephalins are powerful therapeutic analgesics that can readily cross BBB (14).

This work suggests certain compounds as carriers of L-Dopa that will help it to successfully cross BBB without the apprehension of decarboxylation and help enhance its brain exposure. We have considered cyclic peptides and cyclic peptidomimetic compounds of appropriate size to carry L-Dopa. Cyclic peptides and peptidomimetic compounds serve us with an excellent opportunity to have a carrier with all hydrophobic groups disposed outwards and feasibility to anchor drug inside in a masked fashion.

## METHODOLOGY

Ab initio Hartree Fock molecular orbital calculations (15) with complete geometry optimization (16) have been carried out first on cyclic peptide (empty carrier), then on L-Dopa cyclic peptide complex (carrier with L-Dopa). 6-31G basis set (17) has been used throughout. L-Dopa is not covalently linked to carrier. It is only being “transported” through non bonded interactions by carrier. Reorganization required in transporter to carry L-Dopa and reorganization required in L-Dopa to comfortably fit inside transporter have both been calculated. Interaction energy between L-Dopa and carrier is calculated as

Interaction energy = E_complex_ – (E_L-Dopa_ + E_carrier_)

Interaction energy is a measure of overall feasibility of L-Dopa carriage by that carrier. All calculations have been performed utilizing GAUSSIAN ’03 (18) software. GAUSSVIEW (19) has been used for all graphical purposes. Interaction energy calculations will help in analyzing and predicting whether drug will remain held in carrier until delivery at target or there are chances of drug being expelled before reaching target. How difficult it would be to deliver drug non enzymatically at target? Such querries are difficult to answer in absence of accurate carrier-drug interaction calculations. Since the carrier suggested is a cyclic peptidomimetic compound we have also performed relevant calculations to explore its self aggregation tendency. Only monomeric form of carrier can undergo passive diffusion. Therefore, it is important to ensure that suggested peptidomimetic compound does not undergo nanotube formation. Intermolecular interaction calculations to highlight this aspect are also presented here.

## RESULTS AND DISCUSSION

The basic concept behind these computations is that overall favourable interactions between L-Dopa and carrier along with low reorganization energies for both that is, carrier and drug; will allow L-Dopa to be transported by proposed compound. In addition to the above, carrier must possess required hydrophobicity and molecular weight low enough to cross BBB so that it can deliver drug at target. This has been achieved by systematically toning down the size of carrier and converting backbone from peptide to peptidomimetic. All peptide linkages have been converted to hydrophobic backbone leaving only a few essential ones required to anchor drug molecule.

**Figure 1:**
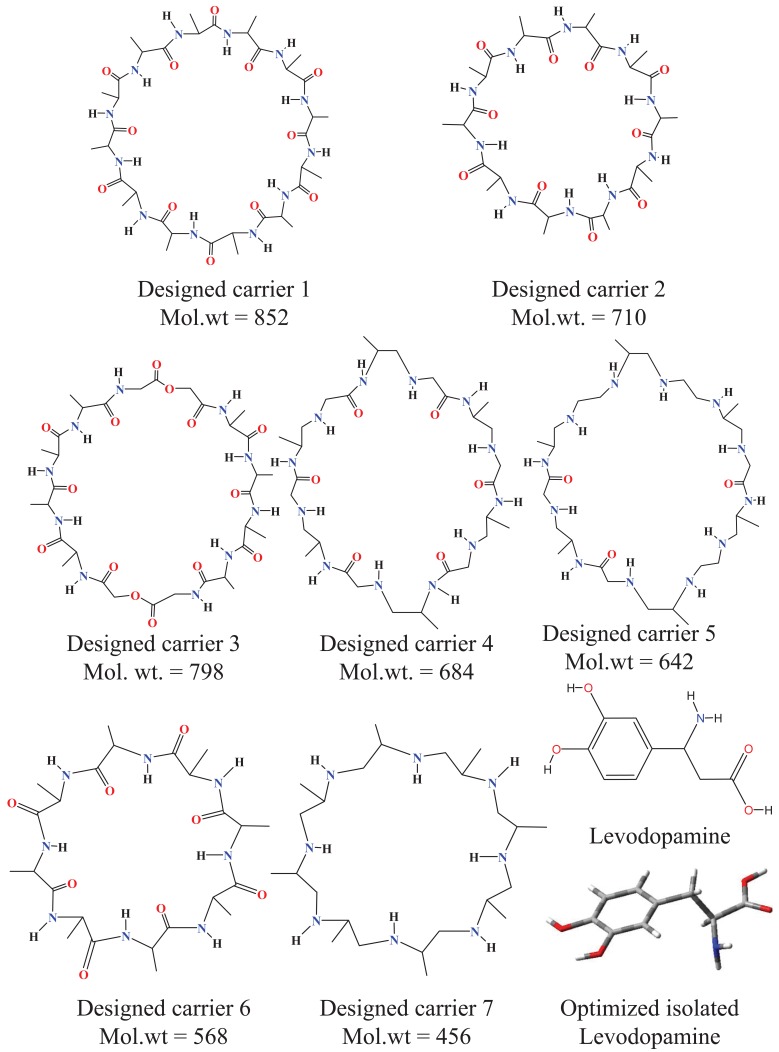
Chemical structures for designed carriers and drug to be carried.

**Figure 2:**
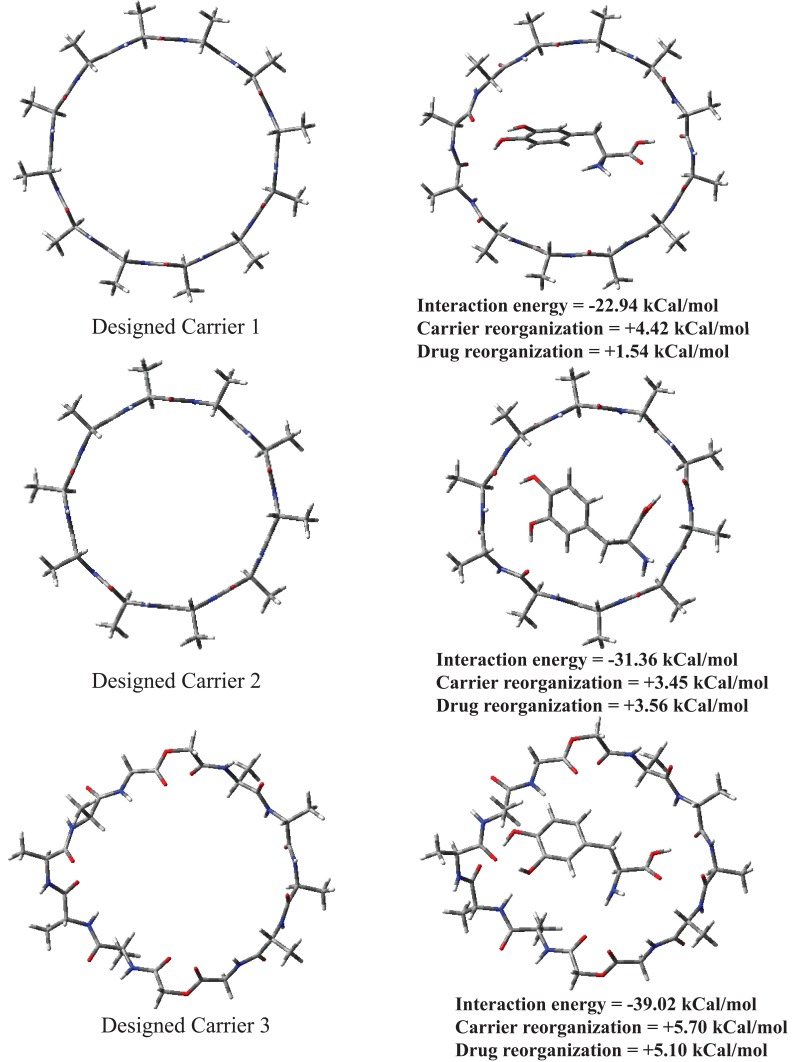
Optimized conformations of empty carriers and when complexed with L-Dopa.

**Figure 3:**
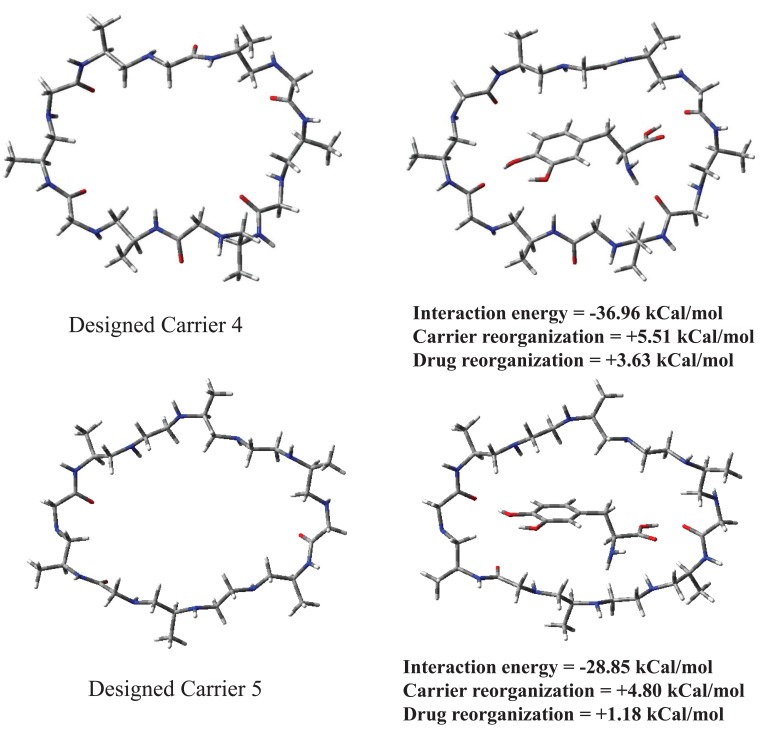
Optimized conformations of empty carriers and when complexed with L-Dopa.

Chemical structures for designed carriers and drug to be carried are shown in Fig. 1. Conformations of empty carriers as well as in complex with L-Dopa after complete geometry optimizations are shown in Figs 2 and 3. Reorganization energies are shown which are a measure of changes in conformation of drug or carrier to form efficient complex. There is a minor change in conformation of all designed carriers after complexing L-Dopa. This implies ease of carriage as carrier reorganization required to carry L-Dopa is quite low (<8 kCal/mol) as needed. L-Dopa reorganization as compared to isolated drug conformation (c.f. Fig. 2) is negligible. L-Dopa carriage feasibility by each designed carrier can be assessed in terms of overall interaction energy. However, main emphasis is on molecular weight in desired range and hydrophobicity required to cross BBB by passive diffusion. Peptide backbone was used in carrier 1 and 2 with smallest hydrophobic substituent to first understand size of backbone required. Carrier 1 is larger than required to carry L-Dopa. Carrier 2 can comfortably accommodate L-Dopa with enhanced interaction due to closer fitting. Now keeping size similar to carrier 2; different peptidomimetic backbones were considered. Designed carrier 3 contains mixed peptide and ester backbone with ester linkages only at few places to enhance affinity of carrier towards L-Dopa. Indeed, it shows enhanced affinity for L-Dopa but at the cost of increased molecular weight. To keep a balance of hydrophobic and polar backbone, designed carrier 4 contains alternating peptide and (-CH_2_-NH-) backbone. Interaction energy predicted shows that converting backbone to partially hydrophobic does not significantly alter carrier’s affinity for L-Dopa as long as anchoring points for L-Dopa are provided. Designed carrier 5 utilized hydrophobic backbone to its maximum leaving polar peptide linkage only at points to anchor L-Dopa. There is reduction in affinity for L-Dopa but still sufficient enough to carry L-Dopa. Strong interaction between carrier and drug is undesirable as it may hinder non enzymatic delivery at target. Change in backbone has led to significant reduction in molecular weight of designed carrier. It is encouraging that carrier and drug reorganization are still quite low. From these points of view peptidomimetic backbone containing ester type linkages mixed with peptide backbone (c.f. carrier 3) is less preferable as opposed to peptidomimetic backbone containing CH2NH linkage mixed with peptide linkages or alone (c.f. carriers 4 and 5). Carrier 5 is recommended as optimum choice. This peptidomimetic backbone is predicted to be reasonably stable in presence of metabolic enzymes (20) and should hopefully enhance delivery of L-Dopa at target. Further reduction in molecular weight of carrier is possible only if L-Dopa is vertically carried inside aggregated form of carrier.

**Figure 4:**
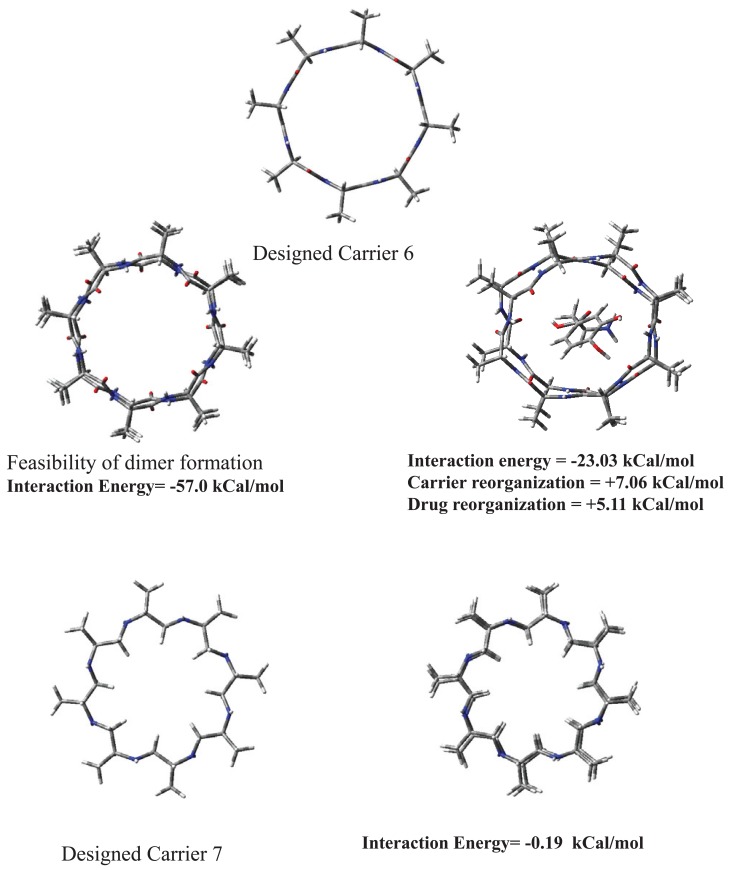
Self aggregation in designed carriers 6 and 7.

Cyclic peptidomimetic compounds possess tendency to self aggregate. Although, CH_2_NH backbone would be less prone to self aggregation as compared to peptide backbone. Considering some possibility of self aggregation we have shown in carrier 6 that self aggregated form can also carry L-Dopa after adjusting backbone size of monomer appropriately. Self aggregation phenomenon is a complex interplay of backbone size, that is, diameter of cyclic peptide, nature of backbone etc. Our calculations in this direction are shown in Fig. 4. Carrier 6 is a small peptide and shows strong self aggregation tendency which slowly dies off after aggregation of about a dozen molecules. Aggregated form can also carry L-Dopa but its molecular weight may not allow passive diffusion through BBB. Carrier 7 does not show aggregation (c.f. Fig. 4) as the largely hydrophobic backbone is not favourable for self aggregation and suggests existence as monomer. It is predicted that carrier 5 (although larger than carrier 7) may thus remain as monomer and should carry L-Dopa efficiently to brain through passive diffusion across BBB leading to enzymatic or non enzymatic delivery of drug thereafter. Synthetic work is in progress to authenticate this research work.

## CONCLUSIONS

Cyclic peptides and peptidomimetic compounds have been studied by ab initio Hartree Fock molecular orbital calculations as possible carriers of L-Dopa to enhance its brain exposure. Computer aided designing has been done keeping in mind target of drug delivery. Small cyclic peptidomimetic compound with CH_2_NH backbone has been suggested as possible carrier of L-Dopa. Suggested carrier is significantly hydrophobic and of appropriate molecular weight to facilitate drug’s significant passive diffusion into brain. Drug will be held inside carrier until delivery at target which may be enzymatic or non enzymatic. Carrier efficiency is predicted to be sufficient so as not to expel drug before delivery at target.
